# Axon Collaterals and Brain States

**DOI:** 10.3389/fnsys.2018.00032

**Published:** 2018-07-17

**Authors:** Kathleen S. Rockland

**Affiliations:** Department of Anatomy and Neurobiology, Boston University School of Medicine, Boston, MA, United States

**Keywords:** axon branching, axon topology, intrinsic collaterals, Meynert neurons, conduction velocity, distributed processing, synchronicity

## Abstract

Multiple mechanisms have been identified as relevant to plasticity, functional stability, and reliable processing across brain states. In the context of stability under “ever-changing conditions” (this Topic), the role of axons has been relatively under-investigated. The highly branched topologies of many axons, however, seem well designed to differentially recruit and regulate distributed postsynaptic groups, possibly in a state-dependent fashion. In this Perspective, I briefly discuss several examples of axon collateralization, and then some of the branch-specific features that might subserve differential recruitment and whole brain activation. An emerging principle is that the number of collaterals and number of target structures are not stereotyped. Rather, axons originating from one defined source typically send branches to diversified subsets of target areas. This could achieve heterogeneous inputs, with different degrees of synchronicity. Variability of neuronal responses has been suggested as inversely proportional to the degree of temporally correlated input. Increased input homogeneity, driven by sensory stimulation or behavioral conditions, is reported to reduce neuronal variability, with axon collateralization potentially having an important role.

Changes in brain state are associated with microcircuitry changes in neuronal firing properties and with macro-level changes in synchronous or asynchronous patterns of brain activation. In this framework, axons been relatively less investigated (Barry, 2015), and then mainly in the context of conduction velocity of action potentials and the increased alertness that can result from changes in conduction velocity (e.g., Stoelzel et al., 2017). In addition, however, the highly branched topology of many axons seems well designed to differentially recruit distributed postsynaptic neuronal groups, possibly in a state dependent manner. Signal processing can be effected in at least three axonal domains; namely, mapping, amplification, and timing (Innocenti et al., 2016; Innocenti, 2017). In this Perspective, I briefly discuss first, network examples of axon collateralization, and then some of the axon-intrinsic features that might underlie differential postsynaptic recruitment. Lacking detailed parameters or mechanisms, the goal is mainly to highlight general features that might figure in the control and transitions of brain states.

## Axon collaterals

All axons have an elaborate distal arborization in the target structure. Many axons in addition have multiple branches (aka, collaterals) which target distinctly different structures (reviewed in Rockland, 2013, 2018). A prime example is the branching of axons from layer 5 corticothalamic neurons. This has been repeatedly documented and is often discussed as a key mechanism by which an organism can distinguish if changes in sensory input are produced by changes in the environment or by self-initiated movements (“efference copy” or “collateral discharge,” Guillery and Sherman, 2011).

In rodents, where there is a large number of applicable techniques for investigating this issue, collateralization is known to be relatively common. To give several examples: (1) neurons in the hippocampal subiculum project to the mammillary bodies, the retrosplenial cortex, or by collaterals to both (rat: Kinnavane et al., 2018); (2) serotonergic neurons in the dorsal raphe (DR) nucleus contact *in various combinations* the striatum, prefrontal cortex, and amygdala (rat: Gagnon and Parent, 2014) as well as combinations of autonomic nuclei (Waselus et al., 2011). In this case, activation of the stress response by DR collaterals might achieve synchronized activation of nuclei associated with neurohormone release or pressor responses. Differential, coordinated activations of forebrain structures may contribute to the multifaceted but related DR functions, such as regulation of the sleep-wake cycle, modulation of pain signals, or mood expression (Gagnon and Parent, 2014).

Third, anatomical studies of mouse thalamocortical projections identify “multispecific axons” that branch widely to restricted domains in separate cortical (and subcortical) areas. These have been hypothesized to orchestrate the fast emergence and reconfiguration of spatially distributed, synchronizable neural assemblies (Clasca et al., 2016).

Fourth, a recent study of corticocortical connections using whole-brain axonal tracing in mouse visual cortex found that 23 of 30 neurons contacted from two to seven other cortical areas. In confirmation of this result, high-throughput DNA sequencing of genetically barcoded neurons found 44% of 533 neurons to be multiply projecting (“broadcast neurons,” Han et al., 2018). Han et al. provisionally distinguished two broad types of projecting neurons, a smaller “dedicated” (uni-target) subpopulation, co-existing with a prevalence of “broadcasting” (multiply projecting) cells. Could this architecture subserve modulations in cognitive state and sensory processing?

## Non-stereotyped collateralization

As remarked above, neurons that collateralize do so in a non-stereotyped pattern. Within a designated projection (defined by the origin), neurons send branches to a diversified subset of target areas (“*in all combinations*”). This observation is so consistent that it can be considered a rule, although the detailed parameters have not been tabulated. The functional significance is also unclear, but emerging results suggest that the heterogeneous and variable inputs to a cortical neuron (and, by extension, we might infer to neural assemblages) are important in driving variability and spike train changes across experimental trials (Gomez-Laberge et al., 2016 and see below).

## Intrinsic collaterals

In all species, long distance cortical projection neurons, in addition to single or multiple extrinsic targets, typically have an elaborate intrinsic arborization. Some cortical neurons have only an intrinsic (local) collateralization, and some only an extrinsic (rat: Kita and Kita, 2012). Although there are only scant data for the actual proportions of intrinsic and extrinsic arborizations (Parent et al., 2000; Rockland, 2018), evidence suggests that this will be highly variable. Even within the system of intrinsic connections, not only are there differences in number of collaterals and number of synaptic boutons, but a single neuron can have a mix of myelinated and unmyelinated collaterals (cat visual cortex: Martin et al., 2014; Koestinger et al., 2017). Branch specific myelination could result in increased, branch specific conduction velocity, although Koestinger et al. suggest it may have more to do with factors like increased security of transmission, presumably, again, branch-specific.

A curious observation related to myelination pertains to the stria of Gennari, the myelinated band of axons in layer 4B of primate area V1. Since this consists of intrinsic collaterals, the common explanation, that myelination is a means of increasing conduction velocity over long distances, is not immediately applicable. Local collaterals do not need (?) augmentation in relation to distant targets and in fact, for the synchronicity one might have predicted, enhanced local conduction (via myelination?) seems paradoxical. Could it be that the myelination relates to other factors, such as plasticity-related (or state-related) changes in axon diameter?

## An interesting example: meynert cells in area V1 of nonhuman primates

**Figure 1:**
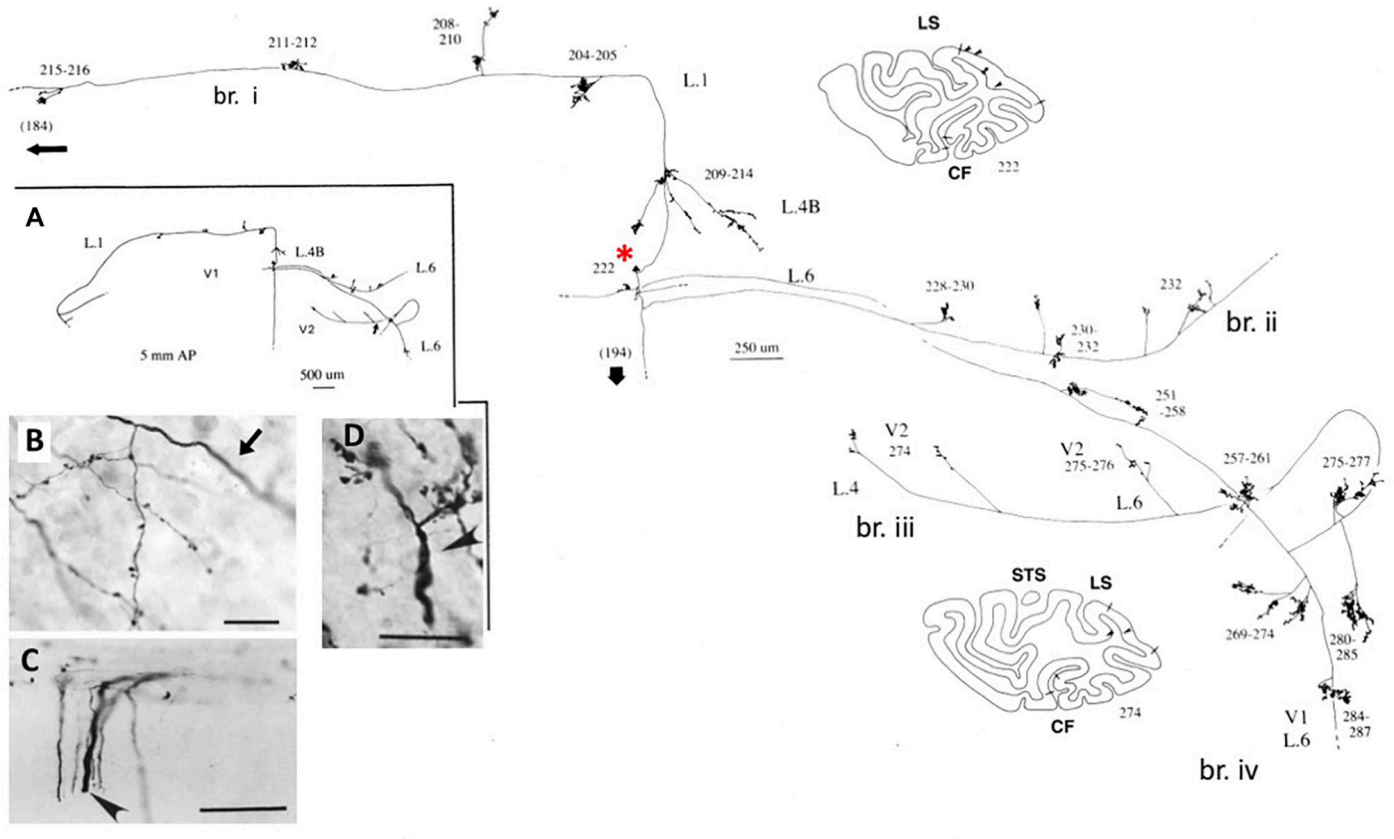
A typical, spatially extended proximal axonal arborization of a Meynert cell (red asterisk) in primary visual cortex of a macaque monkey. There are three major intrinsic collaterals (labeled as br. i, ii, iv), extending 3.0 mm dorsal in layer 1, 3.0 mm ventral in layer 6, 4.0 mm ventral in layer 6, and 0.5 mm in layer 4B, as indicated by arrowheads in the two coronal section outlines (sections 222 and 274, where dorsal is to the left). All together, the intrinsic collaterals span 5.9 mm anterior-posterior (117 sections × 50 μm). A further, extrinsic collateral (br. iii) occurs in layers 4 and 6 of area V2. Portions of the individual collaterals and of the main axon (thick arrow) could not be followed, as is indicated by dashed lines. The low magnification inset (**A**, at left) provides a schematic overview of the general configuration. Numbers denote individual sections, where 20 numbers = 1.0 mm. All branches have numerous small synaptic clusters, one of which is illustrated in **(B)**. Note diminished diameter between the main axon (arrow) and the terminal arborization. Extrinsic axons **(C)** are of variable diameters (one large diameter axon at arrowhead). **(D)** Extrinsic terminations in area MT include some large diameter axons. Scale bar = 25 μm in **(B)**, 100 μm in **(C)**, 20μm in **(D)**. CF, calcarine fissure; LS, lunate sulcus; STS, superior temporal sulcus; L, layer. Modified from Figures 1, 9 in Rockland and Knutson (2001) and Figures 6f, 7b from Rockland (1995) with permission.

These large neurons, at the border of layers 5 and 6, variably project to extrastriate area MT, and/or other visual association areas, and/or to the pulvinar nucleus, and/or to the superior colliculus and pretectum (Weisenhorn et al., 1995; Rockland and Knutson, 2001). The intrinsic collateralization is exceptionally extensive, measured as 8.0 mm from the soma, on the basis of subtotal reconstructions, and having at least 800–1,370 boutons (Figure 1). Across the axonal arborization, there appears to be a distinct variability in branch diameter; that is, extrinsic branches directed to area MT are large (diameter ~3.0 μm), but the intrinsic branches and those projecting to the pulvinar and superior colliculus appear smaller, as judged by light microscopy (cf. Figure 1B (intrinsic) and Figure 1D (extrinsic to area MT). Varying diameters presumably indicate differences in the degree of myelination and, by inference, in conduction velocity.

## Functional significance of axon branching

**Figure 2:**
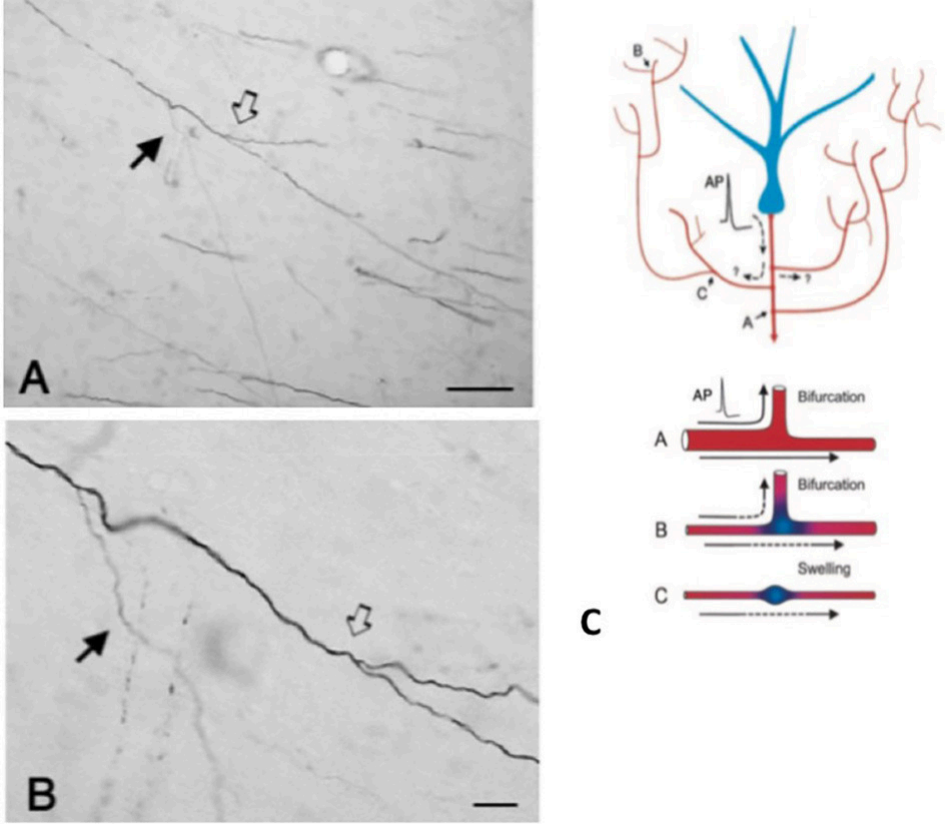
A typically branched segment of axon in the white matter (macaque monkey). The segment originates from a neuron in parietal cortex and is seen here in the vicinity of ventral temporal cortex. Panel **(A)** is lower magnification of **(B)**. Note double bifurcations, where the first daughter branch (solid arrow) is conspicuously thinner (and unmyelinated?) than the main axon. In the second, slightly more distal bifurcation (hollow arrow), the daughter branches appear about equal in diameter, but both are thinner than the main axon. Scale bars = 100 μm **(A)** and 10 μm **(B)**. Reproduced from Zhong and Rockland (2003) with permission. **(C)** Schematic of a neuron (blue) and its extended branching topology (foreshortened for the sake of convenient formatting). An action potential (AP) can follow circuitous paths **(A–C)** to multiple targets. Reliability of propagation depends both on active electrical properties of the axon and its geometry, including membrane inhomogeneties such as swellings and incompatible branch diameters. Below: schematic to illustrate reliable propagation (**A**, with optimal impedance matching between the mother and daughter branches), and slowed or failed propagation (**B**, where the daughter branch has an enlarged diameter; **C**, where there is an interposed membrane swelling). Reproduced from Huguenard (2000) with permission.

An important component of collateralization is that the daughter branches are often not uniform, but especially at branch points, vary in diameter (Figure 2). Variability in diameter together with other parameters will impact on excitability, conduction velocity, and other aspects of signal propagation. Other impacting parameters include width of myelin and intermodal length, and density and distribution of ion channels (reviewed in Debanne et al., 2011; Seidl, 2014; Bucher, 2016; Seidl and Rubel, 2016; Rama et al., 2018). These would have effects on neural response properties at the microcircuitry level. At the more global level, summed activity of multiple projecting axons, with varying conduction velocities across an interlinked network, could result in a spectrum of synchronous and/or asynchronous activations (Mitra et al., 2015; Zeki, 2016). Differential recruitment of postsynaptic populations or network recombinations could be factors in state transitions or modulation.

The axon geometry, active electrical properties, and membrane inhomogeneities at branch points are well-known as factors of reliable propagation (e.g., Manor et al., 1991; Innocenti et al., 1994; Tettoni et al., 1998; Huguenard, 2000; Ofer et al., 2017). This leads to several different scenarios about temporal features consequent to collateralization.

First, there can be synchronous activation throughout the daughter branches. The auditory brainstem pathways project via a single bifurcating axon to ipsi- and contralateral targets (respectively, short and longer physical pathways). Isochronic transmission is achieved by differential myelination and axon caliber of the two daughter branches (i.e., smaller caliber and shorter intermodal lengths ipsilaterally; Seidl, 2014; Seidl and Rubel, 2016). Since most branched axons, in comparison with brainstem auditory pathways, cover a larger territory and subserve less well defined functions, data are largely incomplete or lacking for other systems. One might predict, however, in the case of synchronicity, that proximal branches (i.e., the intrinsic collaterals of Meynert or other pyramidal cells) would have anatomical specializations resulting in longer conduction times, to compensate for and match with the longer distances of extrinsic collaterals. As noted above, this simple prediction does not seem to hold. Further investigation will entail sampling from identified axons over long distances, and would not be easy to achieve.

Second, branch-specific activation may be asynchronous. This could be due to selective failure of transmission and/or asynchronous conduction times across the axon arbor (Figure 2; Huguenard, 2000; Bucher, 2016). Models of cortical circuits describe distinctive routing states of short-lived transient synchrony that could dynamically shape the flow of information (Palmigiano et al., 2017). Comparable experimental data are largely lacking for long distance axons. The collateral topology of thalamocortical projections, however, provides one example evocative of a complicated, activity- and/or state dependent asynchronous activation pattern.

Cortical and thalamocortical activity is highly state-dependent; and the interaction of presynaptic extrinsic inputs (branch specific?) with intrinsic membrane and synaptic properties of postsynaptic neurons is considered fundamental to the generation of rhythmic activity (with “wide ranging effects from enhancing or blocking sensory-motor processing….,” McCormick et al., 2015).

Variability in cortical responses is paradoxical since these also serve as substrates of stable sensory experience. Neuronal variability has been associated with the degree of heterogeneous synchrony across extrinsic input; that is, sensory stimulation or behavioral conditions that increase input homogeneity to a given area is predicted to also *reduce* neuronal variability (Gomez-Laberge et al., 2016). Recent discussions of microcircuitry responses have speculated about a prominent role of slight variances or difference in information: “But what if the differences between the connectivity within cohorts of cells of the same class [aka, presynaptic axonal inputs] are important to circuit function?” (Morgan and Lichtman, 2017).

## Dynamic axon properties

Changes in response latency have been reported in relation to different states of alertness. In the corticothalamic pathway, increased alertness results in significantly shortened response latency. This and /or changes in firing frequency of arriving impulses may be responsible for a dramatically increased response reliability for the subpopulation (58%) of visually responsive corticogeniculate neurons (in rabbits: Stoelzel et al., 2017). These results pertain to physiologically identified single axons; but one can speculate about a wider applicability to branches of collateralized axons.

Ongoing processes of synaptogenesis and distal axon turnover have been demonstrated in the adult cortex (NHP: Stettler et al., 2006). At shorter time scales, superresolution microscopy of unmyelinated GFP-labeled CA3 hippocampal in organotypic brain slices shows that axons gradually widen after bouts of high frequency firing, an observation confirmed by electrophysiological recording (Chereau et al., 2017). Other, branch-specific changes are likely to be discovered; for example, terminal arborizations of individually labeled axons from the dorsal raphe have a target-specific percentage of boutons that contain the protein VGLUT3 (larger percentage for branches terminating in the striatum than in the motor cortex). This implies a complex, nonuniform trafficking mechanism across collaterals (Gagnon and Parent, 2014).

## Conclusion

In this Perspective article, I have discussed axon branching as relevant to changes in brain state, with impact effected via branch-specific properties, differential recruitment of postsynaptic ensembles, and whole brain patterns of synchronization. This builds on long-term discussions concerning axonal branching topologies and how these could modulate information processing by time delays in impulse propagation, differential branch-specific filtering, and activity-dependent excitability (e.g., Segev and Schneidman, 1999). With only a few exceptions, such as the auditory brainstem pathway, hard data are still largely lacking about synchronous and asynchronous activations through daughter branches and how these temporal relationships might impact on postsynaptic neuronal responsiveness (but, see Gomez-Laberge et al., 2016; Stoelzel et al., 2017). Thus, a continuing challenge is to elucidate branch-specific features within individual axons and the effects on postsynaptic ensembles. Recent work brings to the fore additional questions about network heterogeneity, including why neurons from a single source area variably project to one or more targets in what is repeatedly being described as “in all combinations.”

## Author contributions

The author confirms being the sole contributor of this work and approved it for publication.

### Conflict of interest statement

The author declares that the research was conducted in the absence of any commercial or financial relationships that could be construed as a potential conflict of interest.
